# Remarks on thyroid malignity in up-to-date category III of the Novel Bethesda System for Reporting Thyroid Cytopathology in thyroidology

**DOI:** 10.1590/1806-9282.20250575

**Published:** 2025-10-27

**Authors:** Demet Sengul, Ilker Sengul

**Affiliations:** 1 Giresun University, Faculty of Medicine, Department of Pathology – Giresun, Turkey.; 2 Giresun University, Faculty of Medicine, Division of Endocrine Surgery, Thyroidology Unit – Giresun, Turkey.; 3 Giresun University, Faculty of Medicine, Department of General Surgery – Giresun, Turkey.

Dear Editor,

The novel 2023 Bethesda System for Reporting Thyroid Cytopathology (TBSRTC) has announced up-to-date recommendations in thyroidology and thyroid cytopathology^1-10^. We are writing to you with interest regarding the article entitled "The Incidence of Thyroid Cancer in Bethesda III Thyroid Nodules: A Retrospective Analysis at a Single Endocrine Surgery Center" by Hassan et al.^11^ This retrospective cohort analysis investigated the risk of malignancy in thyroid nodules with category III, TBSRTC, based on the outcomes of histopathology at a single endocrine surgery center between January 2020 and March 2024. The authors reported that sonography-guided fine-needle aspiration was performed via a 21-gauge (G) needle, utilizing local anesthesia. The study presents several noteworthy findings. First, it proclaimed a high malignancy rate of 33.5% in the nodules with category III, TBSRTC. The authors suggested that this elevated rate might be due to referral and selection bias^11^. Second, would harnessing both the novel terminology for subdivisions of category III of the current TBSRTC, 3rd edition, separately to all the studied and targeted purposes of this work, alter the study's relevant outcome(s)?^4-8^ Third, the study exhibited a significant gender difference in the nodules with category II, TBSRTC, with females exhibiting a 21.2% malignancy rate compared to only 3.4% in males, while females with malignancy had prognostically superior non-invasive tumors^9^. Fourth, would the utilization of thicker or finer needles transmute^12-14^. the desinence(s) of this study? Fifth, this valued work was of a retrospective nature, conducted at a single center, with a lack of molecular testing data due to insurance and technological limitations. Last but not the least, the relatively small size of the category III cohort is also ponderable as another limitation of this study. However, the authors rightly emphasized the essentiality of individualized risk assessments considering gender-specific factors. Further prospective studies with larger sample sizes from diverse geographical regions, incorporating genetic and biological research, are necessary to validate these findings and refine risk assessment strategies for thyroidologists. This issue merits further investigation. We thank Hassan et al.^1^ for their study on .category III, TBSRTC, for thyroidologists.

## Data Availability

The datasets generated and/or analyzed during the current study are available from the corresponding author upon reasonable request.

## References

[1] Sengul D, Sengul I (2023). World Thyroid Day 2023 in thyroidology: no overlook thyroid dis-eases to opt for "thyroid health" purposes. Rev Assoc Med Bras (1992).

[2] Sengul I, Sengul D (2021). Emphasis on the novel age cutoff, 55 years, for postsurgical adjuvant radioiodine as consideration for American Thyroid Association ¾ low-intermediate risk differentiated thyroid carcinoma. Rev Assoc Med Bras (1992).

[3] Sengul I, Sengul D (2022). May 25-31, international thyroid awareness week & May 25, world thyroid day, 2022: indetermination of indeterminate cytology, AUS/FLUS, FN, SUSP, in thyroidology?. Sanamed.

[4] Sengul I, Sengul D (2023). The 2023 Bethesda system for reporting thyroid cytopathology: novi sub sole, subdivision is no more debatable, in thyroidology. Rev Assoc Med Bras (1992).

[5] Sengul I, Sengul D (2024). Evangely, the subcategorization has been announced in the 2023 Bethesda system for reporting thyroid cytopathology: let bygones be bygones in thyroidology!. Rev Assoc Med Bras (1992).

[6] Sengul I, Sengul D (2021). Focusing on thyroid nodules in suspense: 10-15 mm with repeat cytology, Category III, the Bethesda System for Reporting Thyroid Cytopathology, TBSRTC. Rev Assoc Med Bras (1992).

[7] Sengul I, Sengul D (2021). Blurred lines for management of thyroid nodules in the era of atypia of undetermined significance/ follicular lesion of undetermined significance: novel subdivisions of categories IIIA and IIIB in a possible forthcoming The Bethesda System for Reporting Thyroid Cytopathology, 3rd edition; amending versus unnecessary?. Rev Assoc Med Bras (1992).

[8] Sengul D, Sengul I (2023). Subdivision of intermediate suspicion, the 2021 K-TIRADS, and category III, indeterminate cytology, the 2017 TBSRTC, 2nd edition, in thyroidology: let bygones be bygones?. Ultrasonography.

[9] Sengul I, Sengul D, Egrioglu E, Ozturk T (2020). Laterality of the thyroid nodules, anatomic and sonographic, as an estimator of thyroid malignancy and its neoplastic nature by comparing the Bethesda System for Reporting Thyroid Cytopathology (TBSRTC) and histopathology. J BUON.

[10] Sengul D, Sengul I, Soares JM (2022). Repercussion of thyroid dysfunctions in thyroidology on the reproductive system: conditio sine qua non?. Rev Assoc Med Bras (1992).

[11] Hassan I, Hassan L, Balalaa N, Askar M, Alshehhi H, Almarzooqi M (2024). The incidence of thyroid cancer in Bethesda III thyroid nodules: a retrospective analysis at a Single Endocrine Surgery Center. Diagnostics (Basel).

[12] Sengul I, Sengul D (2021). Proposal of a novel terminology: minimally invasive FNA and thyroid minimally invasive FNA; MIFNA and thyroid MIFNA. Ann Ital Chir.

[13] Sengul D, Sengul I (2022). Minimum minimorum: thyroid minimally invasive FNA, less is more concept? Volens nolens?. Rev Assoc Med Bras (1992).

[14] Sengul I, Sengul D (2022). Apropos of quality for fine-needle aspiration cytology of thyroid nodules with 22-, 23-, 25-, even 27-gauge needles and indeterminate cytology in thyroidology: an aide memory. Rev Assoc Med Bras (1992).

